# Dose-dependent serological profiling of AdCLD-CoV19-1 vaccine in adults

**DOI:** 10.1128/msphere.00998-24

**Published:** 2024-12-26

**Authors:** Jung Hyuk Lee, Yuna Shin, Kwang-Soo Shin, Ju Yeon Park, Mi Sun Kim, Young-Shin Park, Wuhyun Kim, Joon Young Song, Ji Yun Noh, Hee Jin Cheong, Chang-Yuil Kang, Sang Hwan Seo, Jae-Ouk Kim, Deok Ryun Kim, Nathaniel S. Hwang, Jae Seung Yang, Jerome H. Kim, Byoung-Shik Shim, Manki Song

**Affiliations:** 1 International Vaccine Institute, Seoul, South Korea; 2 School of Chemical and Biological Engineering, Seoul National University, Seoul, South Korea; 3 Research & Development Center, Cellid Co. Ltd., Seoul, South Korea; 4 Division of Infectious Diseases, Department of Internal Medicine, Korea University College of Medicine, Seoul, South Korea; 5 Interdisciplinary Program in Bioengineering, Seoul National University, Seoul, South Korea; 6 BioMAX Institute, Seoul National University, Seoul, South Korea; 7 College of Natural Sciences, Seoul National University, Seoul, South Korea; Johns Hopkins University Bloomberg School of Public Health, Baltimore, Maryland, USA

**Keywords:** systems serology, SARS-CoV-2, adenovirus vector-based vaccine, effector function, spike protein

## Abstract

AdCLD-CoV19-1, a chimeric adenovirus-based severe acute respiratory syndrome coronavirus 2 (SARS-CoV-2) vaccine, was previously reported to elicit robust antibody responses in mice and non-human primates after a single dose. In this study, we conducted a systems serology analysis to investigate changes in humoral immune responses induced by varying doses of the AdCLD-CoV19-1 vaccine in a phase I clinical trial. Serum samples from participants receiving either a low or a high dose of the vaccine were analyzed for antibody features against prototype SARS-CoV-2 spike (S) domains (full-length S, S1, S2, and receptor binding domain), as well as Fc receptor binding and effector functions. While both low- and high-dose vaccines induced robust humoral immune responses following vaccination, the quality of antibody features differed between the dose groups. Notably, while no significant difference was observed between the groups in the induction of most S1-specific antibody features, the high-dose group exhibited higher levels of antibodies and a stronger Fc receptor binding response specific to the S2 antigen. Moreover, univariate and multivariate analyses revealed that the high-dose vaccine induced higher levels of S2-specific antibodies binding to FcγR2A and FcγR3B, closely associated with antibody-dependent neutrophil phagocytosis (ADNP). Further analysis using the Omicron BA.2 variant demonstrated that the high-dose group maintained significantly higher levels of IgG and FcγR3B binding to the S2 antigen and exhibited a significantly higher ADNP response for the S2 antigen compared with the low-dose group. These findings underscore the importance of considering diverse humoral immune responses when evaluating vaccine efficacy and provide insights for optimizing adenovirus vector-based SARS-CoV-2 vaccine doses.

**IMPORTANCE:**

Optimization of vaccine dose is crucial for eliciting effective immune responses. In addition to neutralizing antibodies, non-neutralizing antibodies that mediate Fc-dependent effector functions play a key role in protection against various infectious diseases, including coronavirus disease 2019. Using a systems serology approach, we demonstrated significant dose-dependent differences in the humoral immune responses induced by the AdCLD-CoV19-1 chimeric adenovirus-based severe acute respiratory syndrome coronavirus 2 (SARS-CoV-2) vaccine, particularly against the SARS-CoV-2 spike 2 domain. These findings highlight the importance of assessing not only neutralizing antibody titers but also the quality and functionality of antibody responses when evaluating vaccine efficacy.

## INTRODUCTION

Since the emergence of severe acute respiratory syndrome coronavirus 2 (SARS-CoV-2) in 2019, over a hundred vaccine candidates for coronavirus disease 2019 (COVID-19) have been developed (1). As of August 2023, more than 10 vaccines have received full or emergency authorization from the World Health Organization (WHO) (2). Numerous studies have highlighted the importance of humoral immune responses for protection against SARS-CoV-2, with neutralizing antibodies proposed as correlates of protection (CoP) (3–7). However, despite the emphasis on neutralizing antibodies as critical indicators of immunity against COVID-19, several studies have also focused on non-neutralizing antibodies, which may confer Fc-mediated protection against SARS-CoV-2 infection (8–13). Furthermore, binding antibodies exhibit greater resilience against diverse SARS-CoV-2 variants than neutralizing antibodies (13–15). This resilience is significant considering that non-neutralizing Fc-functional antibodies not only constitute a large portion of the total antibodies binding to the SARS-CoV-2 spike (S) protein (16) but also facilitate protective immunity via Fc-mediated effector functions, such as antibody-dependent neutrophil phagocytosis (ADNP), antibody-dependent cellular phagocytosis (ADCP), antibody-dependent complement deposition (ADCD), and antibody-dependent natural killer (NK) cell activation (ADNKA) by interacting with Fc receptors (FcR) present on various immune cells (17). In line with this, many studies have shown that non-neutralizing antibodies play a pivotal role in protection against various infectious diseases through antibody Fc-mediated effector functions (18–21).

Systems serology is an advanced approach that provides a comprehensive understanding of humoral immune responses in individuals infected with pathogens or immunized with various vaccines by analyzing multiple features, including antibody isotyping and subclassification, FcR binding profiling, and Fc-mediated effector functions (22, 23). Unlike traditional serological assessments, which primarily measure the quantity and neutralizing capacity of antibodies, this approach integrates high-throughput experimental techniques and computational methods to analyze antibody features and functions, allowing for a comprehensive insight into the quality and function of humoral immune responses. Furthermore, insights from systems serology can elucidate the immune CoP, helping identify the antibody features associated with successful vaccination, thereby guiding the design of more effective vaccines (10–13).

Previously, we reported that immunization with a single dose of AdCLD-CoV19, a chimeric adenovirus (Ad5/35) vector-based wild-type SARS-CoV-2 vaccine, induced robust SARS-CoV-2 S-specific antibody responses in mice and non-human primates (24). Moreover, we demonstrated enhanced neutralizing antibody responses against the SARS-CoV-2 pseudovirus and its variants of concern following prime or booster administration of AdCLD-CoV19-1 (25, 26). Subsequently, the efficacy of the vaccine is being evaluated in phase 3 clinical trials following completion of safety and dose selection in phase 1 clinical trials.

We hypothesized that humoral immune responses may differ between groups receiving different vaccine doses and that the resulting serological disparities in antibodies would influence variations in Fc-dependent effector functions. Therefore, we present a systems serology analysis of individuals who received either a low dose or a high dose of the AdCLD-CoV19-1 vaccine in phase 1 clinical trials. The results from the enzyme-linked immunosorbent assay (ELISA) and neutralizing antibody assay, traditional methods for evaluating SARS-CoV-2 vaccine efficacy, showed no significant differences between the two dose groups post-vaccination. However, using systems serology, both univariate and multivariate analyses revealed that levels of multiple antibody features related to the prototype SARS-CoV-2 full-length S (FS) and S2 domains were significantly higher in the high-dose group compared with the low-dose group, while no significant differences were observed in the S1 domain and receptor binding domain (RBD). In addition, we demonstrated that these features indeed influence the functional assay, specifically the ADNP against the S2 domain, resulting in significant differences between the two dose groups. Furthermore, our analysis was expanded to include the Omicron BA.2 variant. Using systems serology, we observed that the high-dose group maintained significantly higher levels of specific antibody features targeting the S2 antigen of BA.2. Notably, these distinct antibody characteristics translated into significantly elevated ADNP phagoscores for the S2 domain of BA.2 in the high-dose group compared with the low-dose group. These findings indicate that while the quantity and neutralizing capacity of antibodies may be similar across different doses, the antibody features and effector functions induced by varying vaccine doses can exhibit notable differences. Therefore, this study offers valuable insights for establishing criteria to evaluate vaccine efficacy in clinical trials.

## RESULTS

### AdCLD-CoV19-1 induces robust humoral immune responses against prototype SARS-CoV-2 in adults

Serum samples from a Phase I non-randomized multicenter study (NCT05047692) were used to comprehensively evaluate the serological profiles elicited by varying doses of the AdCLD-CoV19-1 vaccine (27). Samples were obtained from healthy male and female participants aged 19–64 years, who had not previously been infected with COVID-19 or vaccinated against SARS-CoV, MERS-CoV, or SARS-CoV-2. The study involved two experimental groups, both vaccinated on day 0. Group 1 received a low dose of AdCLD-CoV19-1 (5.0 × 10^10^ viral particles) as a single intramuscular injection (*n* = 20), while Group 2 received a high dose (1.0 × 10^11^ viral particles) using the same administration method (*n* = 20). The serum samples used for analysis were collected on day 0, before vaccination, and on day 28, 4 weeks post-vaccination.

**Fig 1:**
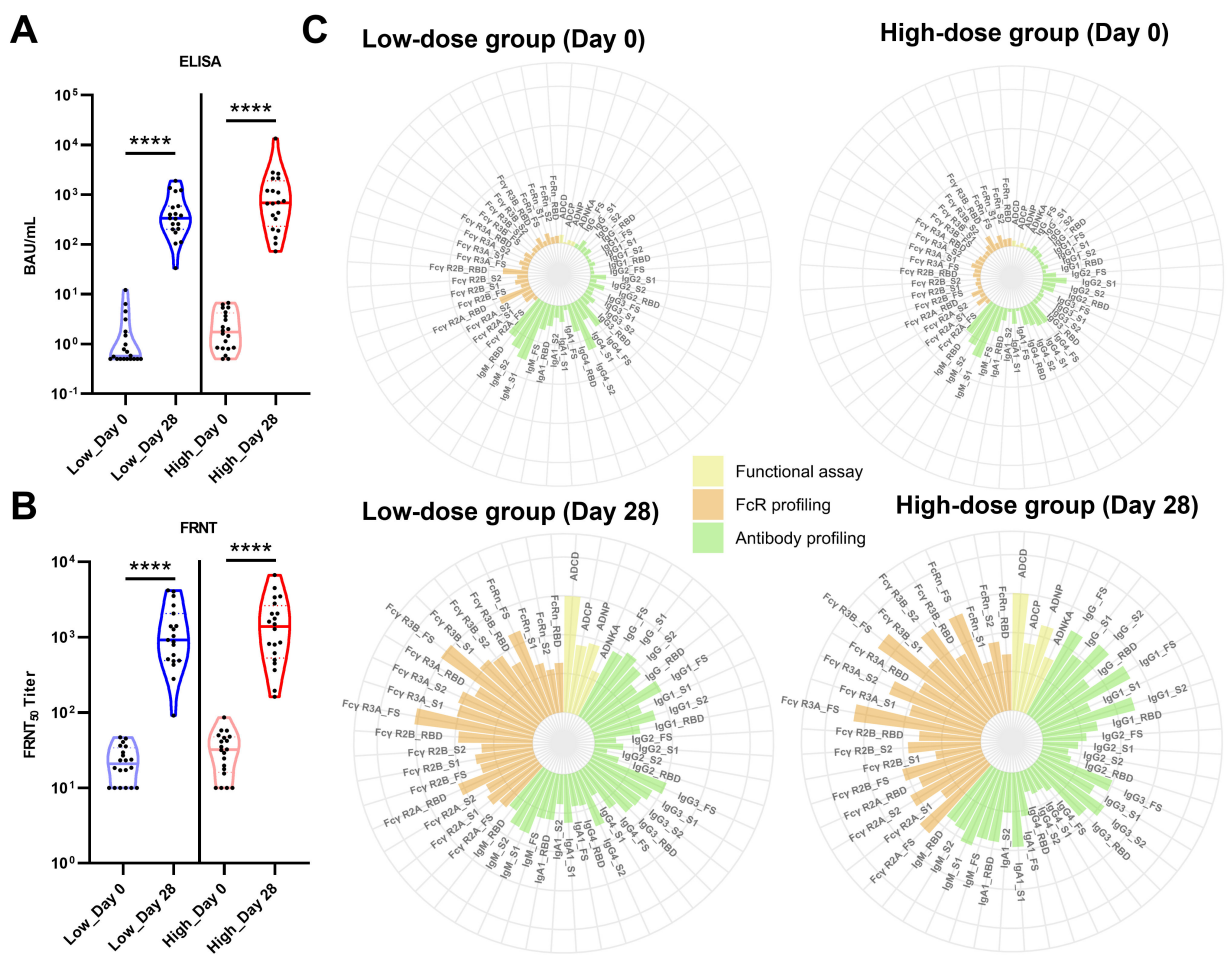
Overall humoral immune responses across the two dose groups post-vaccination. Violin plots (representing medians, interquartile ranges, minima, and maxima) showing the antibody levels in the low- and high-dose groups on days 0 and 28. Individuals were vaccinated on day 0 with two different doses. Dots represent the participants (*n* = 20). (**A**) Anti-SARS-CoV-2 S IgG titers were measured as log10 binding antibody units/mL (BAU/mL) using ELISA. (**B**) Log10 titers of neutralizing antibodies against SARS-CoV-2 measured using the FRNT assay. For the comparison of changes in antibody levels, a Mann–Whitney U test was performed. The *P* value is indicated in the figure. (**C**) Polar plots depicting the mean percentile of prototype SARS-CoV-2-specific antibody features for the two dose groups on days 0 and 28. Percentile rank scores were determined for each antibody feature across all individuals. The colors represent the following feature groups: light-yellow, antibody-dependent functional assays; orange, FcR profiling; and light green, antibody isotypes and subclasses. The SARS-CoV-2 FS antigen was used for the functional assays. SARS-CoV-2 FS, S1, S2, and RBD antigens were used for antibody and FcR profiling. ****P < 0.0001.

Prototype SARS-CoV-2 FS-specific IgG antibody and neutralizing antibody titers were measured using ELISA and a focus reduction neutralization test (FRNT) as the primary endpoint of vaccine efficacy in phase 1 clinical trials (Fig. 1A and B). As expected, both the low- and high-dose groups exhibited a significant increase in IgG antibody titers against the FS protein following vaccination (Fig. 1A). Similarly, neutralizing antibody titers against SARS-CoV-2 were substantially elevated in both dose groups on day 28 compared with those before vaccination (Fig. 1B). However, no significant differences between the two dose groups post-vaccination were observed in either the binding or neutralizing antibody titers despite these antibody titers being slightly higher in the high-dose group than in the low-dose group (*P* = not significant; Fig. S1).

Therefore, we applied a systems serology approach to clinical serum samples from the two groups to comprehensively investigate the humoral immune responses induced by varying doses of the AdCLD-CoV19-1 vaccine, including antibody profiling, FcR binding profiling, and Fc-mediated effector functions, using the prototype SARS-CoV-2 as the antigen (Fig. 1C). Using systems serology, we showed that the overall humoral immune responses were significantly increased by the two varying doses of the vaccine compared with prevaccination, whereas the breadth and quality of antibody features were different between the two dose groups (Fig. 1C). Systems serology analyses revealed robust enhancements in all four functional assays (ADCD, ADCP, ADNP, and ADNKA) against SARS-CoV-2 FS in both dose groups (Fig. 1C; Fig. S2). In addition, a significant increase in antibodies (IgG, IgG1, IgG3, and IgA1) specific to the four SARS-CoV-2 S antigens was observed in both groups (Fig. 1C; Fig. S3A , B, C and F). However, an S-specific IgG4 antibody response was not induced by a single dose of AdCLD-CoV19-1 (Fig. 1C; Fig. S3E). Notably, variations in the increase in antigen-specific IgG2 and IgM were observed in the low- and high-dose groups. While the low-dose group showed a significant increase in the IgG2 response limited to FS and S1, the high-dose group exhibited a significant increase against all four antigens (Fig. 1C; Fig. S3C). Moreover, no considerable increase in IgM levels was observed in the low-dose group, whereas the high-dose group showed a significant increase against the FS antigen (Fig. 1C; Fig. S3G). In FcR profiling, significant increases were observed in all five FcRs (FcγR2A, FcγR2B, FcγR3A, FcγR3B, and neonatal FcR (FcRn)) against the four SARS-CoV-2 domains in both groups (Fig. 1C; Fig. S4). Overall, these results indicate that the AdCLD-CoV19-1 vaccine induces a broad and potent humoral immune response against the prototype SARS-CoV-2 and that varying doses of the adenoviral vector-based vaccine may induce different antibody characteristics.

### AdCLD-CoV19-1 induces distinct antigen-specific antibody features between the low- and high-dose groups

**Fig 2:**
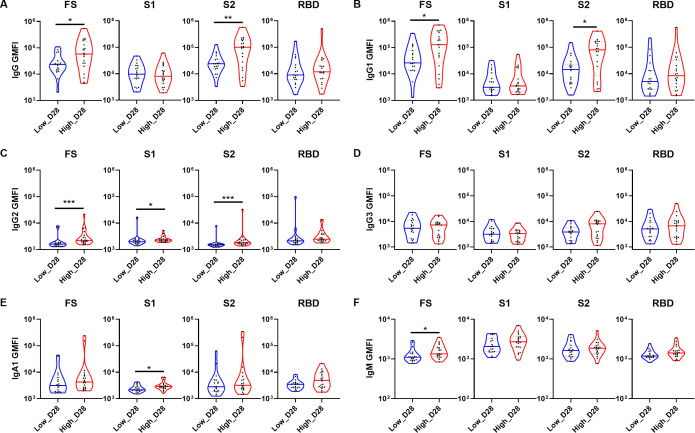
Differences in antibody architecture between the two groups on day 28 post-vaccination. Violin plots illustrating univariate comparisons of antibody profiling specific to prototype SARS-CoV-2 FS, S1, S2, and RBD between the low- and high-dose groups on day 28. Data are presented as geometric mean fluorescence intensity (GMFI). The analyzed antibody isotypes and subclasses are (**A**) IgG, (**B**) IgG1, (**C**) IgG2, (**D**) IgG3, (**E**) IgA1, and (**F**) IgM, which are specific to four different antigens. The two groups were compared using the Mann–Whitney U test. The *P* values are shown in the figure. *P < 0.05, **P < 0.01, and ***P < 0.001.

To further investigate the differences in antibody profiles between the two groups following vaccination, we compared the isotypes and subclasses of antibodies in serum samples collected from the two groups 28 days post-vaccination against four prototype SARS-CoV-2 antigens (FS, S1, S2, and RBD) (Fig. 2). Importantly, the high-dose group exhibited significantly higher levels of IgG and IgG1 antibodies against both the FS and S2 antigens compared with the low-dose group. In contrast, there were no significant differences in antibodies against the S1 and RBD antigens between the two groups (Fig. 2A and B). Additionally, higher levels of IgG2 antibodies against the FS, S1, and S2 antigens were observed in the high-dose group compared with the low-dose group; however, both doses induced modest levels of these antibodies (Fig. 2C; Fig. S3C). Consistent with previous preclinical studies (24–26), the AdCLD-CoV19-1 vaccine predominantly elicited FS-specific IgG1 and IgG3 antibody responses. However, unlike the IgG1 antibody responses, there were no significant differences in IgG3 antibody levels between the two groups (Fig. 2D). For the isotypes, S1-specific IgA1 and FS-specific IgM antibody titers were higher in the high-dose group than the low-dose group (Fig. 2E and F). Interestingly, except for IgG2 and IgA1, no significant differences were observed in antibody features targeting S1 and the RBD between the two dose groups on day 28 post-vaccination. These data indicate that the high-dose group exhibited distinct antibody profiles compared with the low-dose group, particularly against the FS and S2 antigens.

### S2-specific FcR binding profiles differ significantly between the high- and low-dose groups

**Fig 3:**
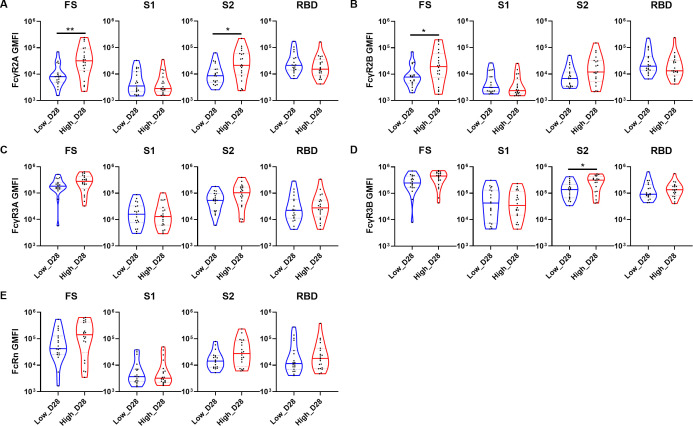
Differences in FcR architecture between the two dose groups on day 28 post-vaccination. Violin plots showing univariate comparisons of FcR profiling specific to prototype SARS-CoV-2 FS, S1, S2, and RBD between the low- and high-dose groups on day 28. FcR binding for (**A**) FcγR2A, (**B**) FcγR2B, (**C**) FcγR3A, (**D**) FcγR3B, and (**E**) FcRn is expressed as GMFI. The two groups were compared using the Mann–Whitney U test. The *P* values are shown in the figure. *P < 0.05 and **P < 0.01.

The interaction between antibodies and FcRs is crucial for inducing Fc-mediated effector functions. Therefore, we compared the FcR binding profiles between the two dose groups at 28 days post-vaccination against four prototype SARS-CoV-2 antigens (Fig. 3). Similar to the antibody features, the high-dose group showed superiority over the low-dose group in terms of overall FS-specific antibody binding to all tested FcRs. Furthermore, it is noteworthy that the high-dose group exhibited higher levels of S2-specific antibodies binding to FcRs compared with the low-dose group, with significant increases observed in S2-specific FcγR2A and FcγR3B binding (Fig. 3A and D). In contrast, the binding of both S1- and RBD-specific antibodies to FcRs was similar between the two dose groups. No significant differences were observed between the two groups in the binding of antibodies to FcRn and FcγR3A, irrespective of the antigens (Fig. 3C and E). These results suggest that distinct FcR binding profiles may be induced depending on the dosage of the adenovirus vector-based SARS-CoV-2 vaccine.

### ADNP response against the S2 domain differs significantly between the high- and low-dose groups

**Fig 4:**
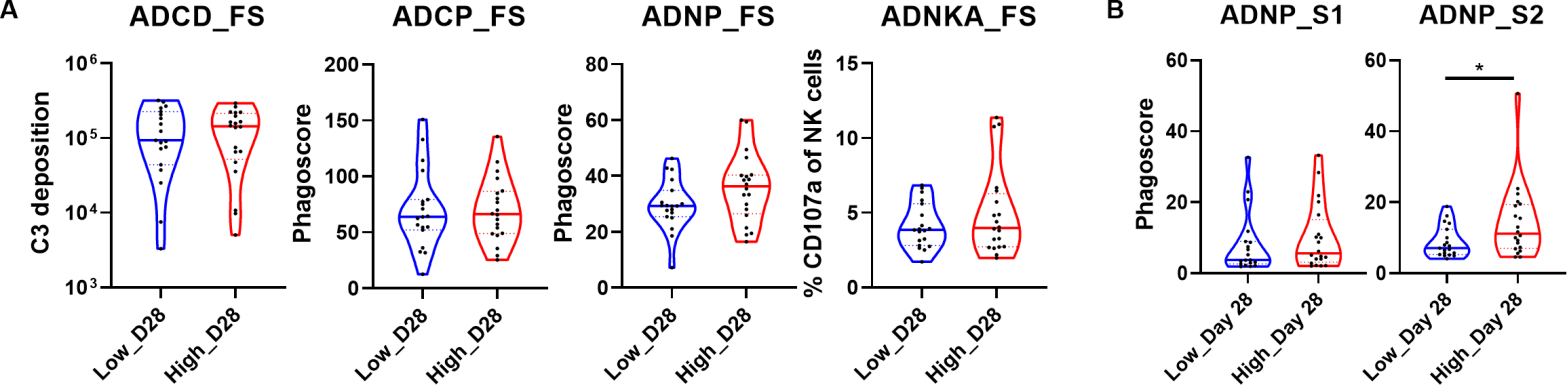
Fc-mediated effector functions of the two dose groups on day 28 post-vaccination. (**A**) Violin plots showing univariate comparisons of four Fc-mediated effector functions specific to prototype SARS-CoV-2 FS between the low-dose and high-dose groups on day 28. Measurements are presented as GMFI for C3 deposition, indicating ADCD; as phagocytosis scores for ADCP and ADNP; and as the percentage of CD107a-positive cells, representing ADNKA. (**B**) The ADNP results for the low- and high-dose groups on day 28 with S1 and S2 antigens are shown as violin plots. The two groups were compared using the Mann–Whitney U test. The *P* values are shown in the figure. *P < 0.05.

We compared various effector functions activated by the interaction between antibodies and FcRs across each dose group 28 days post-vaccination to investigate whether distinct antibody features influenced functional outcomes against the prototype SARS-CoV-2 (Fig. 4). When evaluating the four functional assays for the FS antigen, no significant differences were observed between the two dose groups (Fig. 4A), although the ADNP response was higher in the high-dose group compared with the low-dose group. Given the observed high levels of S2-specific antibodies and their binding to FcγR2A and FcγR3B (Fig. 2 and 3), which are FcRs highly expressed on neutrophils, we investigated whether these antibody features contributed to an increase in the ADNP response. To this end, an ADNP assay was conducted using the S1 and S2 antigens (Fig. 4B). No significant differences in ADNP responses against the S1 domain were detected between the two groups. However, a significantly higher phagoscore for the S2 domain was observed in the high-dose group compared with the low-dose group. Taken together, these results suggest that S2-specific antibodies induced by an adenovirus vector-based SARS-CoV-2 vaccine play an important role in activating Fc-mediated antibody effector functions.

### Low- and high-dose groups show distinct antibody features targeting FS and S2 antigens

**Fig 5:**
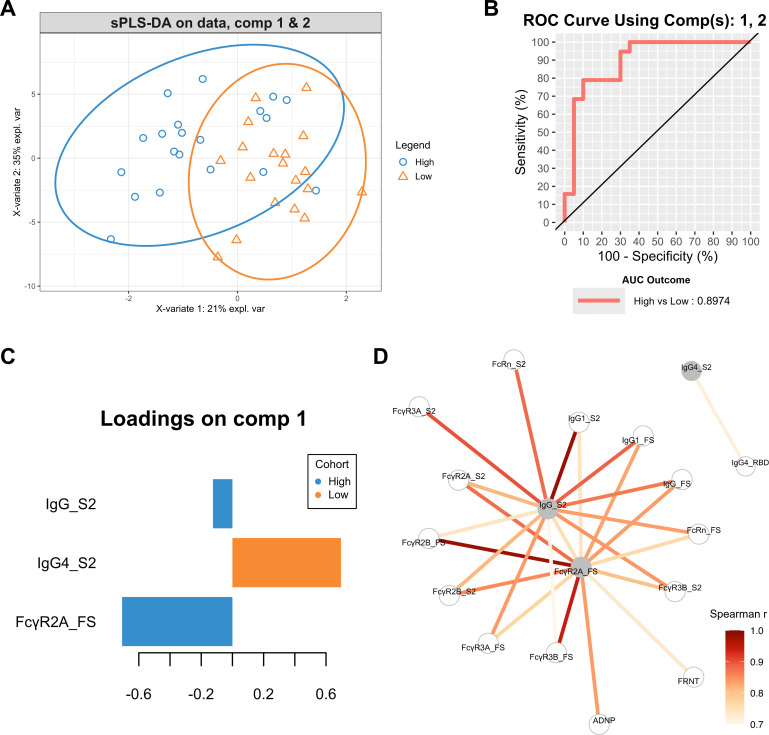
Multivariate analysis of the two dose groups post-vaccination. A sPLS-DA was performed using antibody feature data from the two dose groups on day 28. (**A**) Sample plot with 0.95 ellipse confidence level illustrating the separation of antibody features between individuals in low- and high-dose groups. Colors indicate the dose groups: blue indicates the high-dose group and orange indicates the low-dose group. (**B**) ROC curve and AUC for the model containing components 1 and 2. The AUC value, calculated to compare the two dose groups, was 0.8974. (**C**) The antibody features that significantly contributed to the differentiation between the high-dose group (blue, pointing left) and the low-dose group (orange, pointing right) are shown and ranked by the loading score for component 1. (**D**) A co-correlation network was constructed based on three selected features from the loading plot for X-variate 1 using Spearman’s rank correlation. Only correlations with |*r*| > 0.7 to at least one of the highlighted features in gray are displayed.

To identify the specific antibody features closely associated with each dose group and analyze how effectively these features differentiated the two groups, sparse partial least squares discriminant analysis (sPLS-DA) was conducted based on data from day 28 (Fig. 5A, B and C). Although some subjects from each dose group overlapped in the sample plot, there was a notable difference between the two groups (Fig. 5A). The receiver-operating characteristic (ROC) curve and the area under the curve (AUC) value were used to evaluate the discriminative performance of the sPLS-DA model (Fig. 5B). In the comparison between the two dose groups, the AUC value was approximately 0.89. Within the loading plot based on component 1, two selected variables, FcγR2A for FS and IgG for S2, were associated with the high-dose group, while only IgG4 for S2 was selected for the low-dose group (Fig. 5C). These trends in the multivariate analysis were consistent with previous univariate results, showing that the levels of FcγR2A for FS (Fig. 3A) and IgG for S2 (Fig. 2A) were higher in the high-dose group than in the low-dose group. Although S2-specific IgG4 was selected for the low-dose group, the differences in antibody titers before and after vaccination against all four antigens were not significant in either group (Fig. S3E).

Finally, we conducted a correlation network analysis using the three key features selected from the sPLS-DA to identify additional characteristics that differentiated the low- and high-dose groups (Fig. 5D). The two features associated with the high-dose group were highly correlated with multiple variables for FS and S2 antigens. IgG1, FcγR2A, and FcγR3B specific to the S2 antigen correlated with the two key features of the high-dose group. These newly derived features specific to the S2 antigen have also been verified in previous results, which showed higher values in the high-dose group than in the low-dose group (Fig. 2B and 3C, D). Furthermore, FcγR2A specific to the FS antigen was highly correlated with ADNP for FS. This result may explain the higher phagoscore values observed in the high-dose group compared with the low-dose group against the FS antigen in the ADNP assay, although the difference was not significant (Fig. 4A). Overall, the results of the computational analysis suggest that the antibody features specific to the FS and S2 antigens vary between the low- and high-dose groups. Moreover, the enhanced ADNP response to FS and S2 antigens in the high-dose group may have been driven by these specific antibody characteristics.

### AdCLD-CoV19-1 elicits distinct S2-specific antibody features against Omicron BA.2 variant in the high-dose group

**Fig 6:**
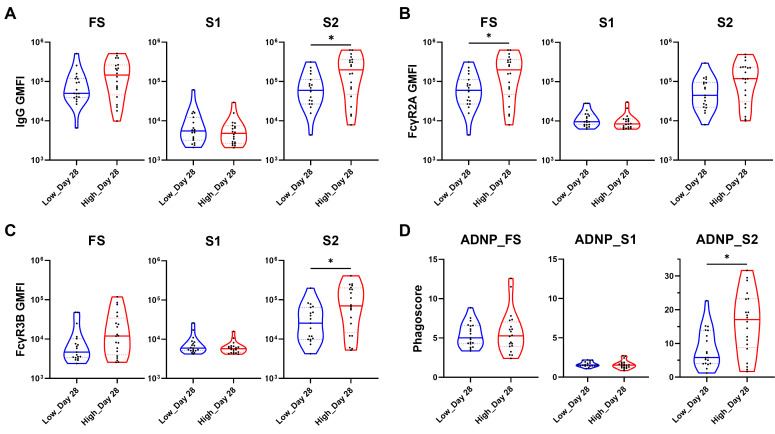
Profiling assays and ADNP of the two dose groups on day 28 post-vaccination against the Omicron BA.2 variant. Violin plots showing univariate comparisons of profiling assays and ADNP specific to Omicron BA.2 FS, S1, and S2 between the low- and high-dose groups on day 28. Antibody features of (**A**) IgG, (**B**) FcγR2A, and (**C**) FcγR3B are expressed as GMFI. (**D**) The ADNP results are presented as phagocytosis scores. The two groups were compared using the Mann–Whitney U test. The *P* values are shown in the figure. *P < 0.05.

To build on the previously observed superiority of the high-dose group in S2-specific antibody features against the prototype SARS-CoV-2, we extended our analysis to variants of concern by conducting systems serology analyses using the Omicron BA.2 variant (Fig. 6; Fig. S5). Specifically, we tested profiling assays for IgG (Fig. 6A; Fig. S5A), FcγR2A (Fig. 6B; Fig. S5B), and FcγR3B (Fig. 6C; Fig. S5C) and ADNP (Fig. 6D; Fig. S5D) against three Omicron BA.2 antigens (FS, S1, and S2), as these features are strongly associated with neutrophil activity and were significantly higher in the high-dose group than in the low-dose group for the prototype SARS-CoV-2 S2 antigen. Similar to the results observed in Fig. 1C, both the low- and high-dose groups exhibited significant increases in all assays for all tested antigens post-vaccination (Fig. S5).

When evaluating the differences between the two dose groups post-vaccination, no significant differences were found between the groups against the S1 antigen (Fig. 6). However, significant differences were observed in antibody features against the FS and S2 antigens. For the FS antigen, FcγR2A levels were significantly higher in the high-dose group compared with the low-dose group (Fig. 6B), while IgG and FcγR3B levels against the S2 antigen were significantly higher in the high-dose group than in the low-dose group (Fig. 6A and C).

Last, we demonstrated that these distinct antibody features in the high-dose group did indeed influence functional outcomes by showing significantly higher phagoscore for the S2 domain in the high-dose group compared with the low-dose group (Fig. 6D). In conclusion, through these experiments, we demonstrated that the distinct S2-specific antibody features observed in the high-dose group were not limited to the prototype SARS-CoV-2 but were also applicable to variants of concern such as BA.2.

## DISCUSSION

In this study, using a systems serology approach, we demonstrated that vaccination with different doses of the AdCLD-CoV19-1 vaccine, a chimeric adenovirus-based wild-type SARS-CoV-2 vaccine, induced distinct antibody features and Fc-mediated effector functions. Both the low- and high-doses of AdCLD-CoV19-1 elicited robust humoral immune responses following vaccination, while no significant difference was observed between the two dose groups, as determined by ELISA and FRNT assays. However, using systems serology, we found significantly higher levels of particular antibody features (IgG, IgG1, FcγR2A, and FcγR2B) specific to the FS antigen in the high-dose group compared with the low-dose group on day 28. These findings are consistent with those reported for the Ad26.COV2.S vaccine from Janssen (28). Importantly, while no significant difference was observed between the two groups in inducing most S1- and RBD-specific antibody features, the high-dose group exhibited higher levels of distinct antibody features (IgG, IgG1, FcγR2A, and FcγR3B) specific to the S2 antigen compared with the low-dose group, which is in line with the previous evidence showing that both S2- and FS-specific FcR binding features were predominantly enhanced features in non-human primates that received the highest dose of the Ad26.CoV2.S vaccine (29). Given that the S2 domain is the most conserved region across CoV strains (30), this finding is supported by the observation that SARS-CoV-2 S2-specific IgG features are associated with cross-reactivity induced by prior exposure to human CoV antigens (31). In addition, antibodies targeting the S2 domain have been shown to be elicited more rapidly following exposure to SARS-CoV-2 than those targeting other domains (32).

Both FcγR2A and FcγR3B are abundantly expressed on the surface of neutrophils (33, 34). FcγR2A on neutrophils has a high affinity for IgG, particularly IgG1, and regulates phagocytosis (35). FcγR3B, the most abundant protein on the surface of neutrophils, binds to multimeric IgG1 and IgG3 but has little to no binding affinity for IgG2 and IgG4 (33). Through these binding interactions, individuals vaccinated with ChAdOx1 nCoV-19 experienced a reduced rate of breakthrough infections for COVID-19 (36). Moreover, S2-specific FcR binding antibodies induced by COVID-19 mRNA vaccination were most actively increased to induce phagocytosis in monocytes and neutrophils during breakthrough infections with variants of concern such as Delta and Omicron (37). In line with this, we found that a single dose of the AdCLD-CoV19-1 vaccine predominantly elicited antigen-specific IgG1 and IgG3 antibody responses. Furthermore, sPLS-DA analysis revealed that both FcγR2A for FS and IgG for S2 were closely associated with the high-dose group. This association was further confirmed by correlation network analysis, which showed a strong correlation between two key features related to multiple variables for FS and S2 antigens, including ADNP for FS.

Notably, in an ADNP analysis involving the S1 and S2 antigens, we found a significantly higher phagoscore for the S2 domain in the high-dose group than in the low-dose group. Furthermore, we extended our analysis to examine the antibody responses against the Omicron BA.2 variant. Similar to the results observed with the prototype SARS-CoV-2 strain, the high-dose group exhibited significantly higher levels of S2-specific antibody features against BA.2, including IgG and FcγR3B levels, compared with the low-dose group. This was associated with enhanced functional activity, with the high-dose group demonstrating a significantly higher phagoscore for the BA.2 S2 domain in the ADNP assay. Recent studies also have shown that ADNP activity strongly correlates with protection against severe COVID-19 and serves as a key predictor of disease outcomes. For instance, ADNP was a critical functional response associated with survival from SARS-CoV-2, as it was more robust in individuals recovering from the virus and aided in clearing the infection (10). Moreover, after receiving the CoronaVac, a whole inactivated virus COVID-19 vaccine, ADNP responses demonstrated cross-reactivity against both the wild-type and Omicron variants (38). In non-human primate studies, ADNP was linked to viral control in the upper respiratory tract against the Omicron BA.4 and BA.5 variants (39).

One limitation of our study is that our results were based on serum samples collected at only two time points: pre- and post-vaccination. Therefore, additional studies are required to assess long-term follow-up serum samples, extending beyond 4 weeks post-vaccination, to evaluate changes in antibody features and immunity when receiving a different vaccine as a booster shot. Nonetheless, the effectiveness of the single-dose AdCLD-CoV19-1 vaccine has been confirmed in various preclinical studies (24–26) and is further substantiated by the identification of heightened immunological responses following the administration of the single-dose vaccine in this clinical study.

Approved adenovirus-vectored COVID-19 vaccines include ChAdOx1 nCoV-19 (AZD1222) from AstraZeneca and Ad26.COV2.S from Janssen. ChAdOx1 nCoV-19 and Ad26.COV2.S utilize viral vectors derived from the chimpanzee Y25 adenovirus and human adenovirus type 26, respectively (40, 41). Several studies have been conducted on the systems serology analysis of these two vaccines (28, 29, 36, 42–44). For instance, humoral immune responses were investigated in humans and non-human primates by varying the dose of the Ad26.CoV2.S vaccine (28, 29, 42). However, these studies predominantly examined the changes in antibody features against the SARS-CoV-2 FS protein and RBD.

In this study, we explored the humoral immune profiles against various SARS-CoV-2 S domains, including FS, S1, S2, and RBD. To the best of our knowledge, this study is the first systems serology investigation to report the differences in antibody characteristics, particularly focusing on the S2 domain, across the vaccinees who received a single dose of AdCLD-CoV19-1. We also demonstrated that high-dose AdCLD-CoV19-1 was superior to low dose in terms of the levels of various immunoglobulins and FcR binding. Moreover, we found that these variables influenced Fc-dependent antibody effector functions, with the high-dose group demonstrating a superior ADNP phagoscore for the S2 domain compared with the low-dose group, not only against the prototype SARS-CoV-2 but also the Omicron BA.2 variant. These findings highlight the importance of considering not only neutralizing antibody levels but also various changes in humoral immune responses from the vaccine dose testing stage onwards, offering insights for optimizing the efficacy of adenovirus vector-based SARS-CoV-2 vaccines.

## MATERIALS AND METHODS

### Study population

Serum samples from the Phase I clinical trial (NCT05047692), which was non-randomized and conducted across multiple centers, were used to thoroughly analyze the serological responses triggered by different dosages of the AdCLD-CoV19-1 vaccine developed against the prototype strain of SARS-CoV-2 (27). Samples were collected between September 9, 2021, and November 5, 2021, from healthy Korean adult males and females aged 19–63 years who had not been previously infected with COVID-19 or vaccinated against SARS-CoV, MERS-CoV, or SARS-CoV-2. All participants provided written informed consent. The study consisted of two experimental groups, both of which received vaccinations on day 0. Group 1 received a lower dose of AdCLD-CoV19-1 (5.0 × 10^10^ viral particles) via a single intramuscular injection (*n* = 20), while Group 2 received a higher dose (1.0 × 10^11^ viral particles) using the same method (*n* = 20). Serum samples were collected on day 0 before vaccination and day 28, 4 weeks post-vaccination. Table 1 summarizes the demographic characteristics of the study participants. These samples were then used to evaluate the antibody responses, neutralization titers, and immune cell reactions induced by the vaccine formulations in the participants of the study.

**TABLE 1:** Demographic characteristics of study participants in the AdCLD-CoV19-1 Phase I clinical trial

	Low-dose group	High-dose group
	(5.0 × 10^10^ VP/dose)	(1.0 × 10^11^ VP/dose)
	*n* = 20	*n* = 20
Gender		
Male, *n* (%)	11 (55.00)	14 (70.00)
Female, *n* (%)	9 (45.00)	6 (30.00)
Age (years)		
Mean ± SD	37.90 ± 12.29	40.75 ± 9.71
Median	36	42.5
Min, max	19.00, 57.00	24.00, 63.00
Height (cm)		
Mean ± SD	169.55 ± 9.42	172.39 ± 7.49
Median	169.35	173.25
Min, max	154.00, 183.90	155.70, 186.50
Weight (kg)		
Mean ± SD	70.75 ± 10.04	70.94 ± 12.89
Median	71.5	69.4
Min, max	56.00, 86.20	51.60, 101.00
BMI (kg/㎡)		
Mean ± SD	24.60 ± 2.81	23.73 ± 3.10
Median	25.1	23.25
Min, max	19.20, 29.70	18.90, 29.90

### Antigen coupling with beads

All antigens used for the analyses were biotinylated (#A39257, Thermo Scientific), desalted with Zeba Columns (#89889, Thermo Scientific), and coupled to the designated beads for each assay. Luminex assays were conducted using four prototype SARS-CoV-2 antigens, including FS protein (#40589-V08H4, Sino Biological), S1 (#40591-V08H, Sino Biological), S2 (#40590-V08H1, Sino Biological), and RBD (#40592-V08H, Sino Biological), as well as three Omicron BA.2 variant antigens, which included FS protein (#SPN-C522b, ACROBiosystems), S1 (#S1N-C52Hv, ACROBiosystems), and S2 (#S2N-C52Hh, ACROBiosystems). These antigens were attached to Luminex Magplex-Avidin microspheres # MA-A012-01, # MA-A013-01, # MA-A014-01, and # MA-A015-01. The complexes were then incubated with blocking buffer (5% BSA in PBS) to be later washed and pooled in assay buffer (1× PBS + 0.1% BSA + 0.05% Tween-20).

Functional assays utilized SARS-CoV-2 antigen-coupled yellow-green fluorescent neutravidin-labeled microspheres (#F8776, Invitrogen) for phagocytosis and red fluorescent neutravidin-labeled microspheres (#F8775, Invitrogen) for complement deposition. The antigen-microsphere complexes were treated with blocking buffer and washed twice with 0.1% BSA (#A3803-100G, Sigma-Aldrich) in PBS (#10010023, Gibco).

### Serum sample preparation

All frozen serum samples were thawed at room temperature, followed by heat inactivation by placing them in heat blocks set to 56°C for 30 minutes. Samples were diluted with PBS and later stored in a freezer at −80°C.

### Enzyme-linked immunosorbent assay

ELISA was performed as previously described (45). Ninety-six-well plates (#3590, Corning/Costar) were coated with the stable trimer form of SARS-CoV-2 S protein (#SPN-C52H9, Acro Biosystems) at a concentration of 2.0 µg/mL in Dulbecco’s phosphate buffer saline (#SH30028.03, HyClone) and incubated overnight at 4°C. The plates were washed four times with 1× wash buffer (PBS + 0.05% Tween 20 (#P1379, Sigma-Aldrich)) per well and then blocked with blocking buffer (#37538, Thermo Scientific) for 2 hours at room temperature. Serum samples were initially diluted 1:20, followed by a threefold serial dilution. After blocking, the plates were washed once, and the diluted samples were added. All plates were incubated for 1 hour at room temperature with shaking. Following the wash step, the plates were incubated with an HRP-conjugate detection antibody solution (#555788, BD Pharmingen) diluted at 1:1,000 in the blocking buffer for 1 hour at room temperature with shaking. After the final wash, 3,3',5,5'-tetramethylbenzidine (TMB) substrate (#5120-0077, KPL) was added to the plates and the reaction was stopped with TMB stop solution (#5150-0021, KPL). The optical density was measured at wavelengths of 450 nm and 650 nm using a microplate reader (Spectramax, Molecular Device). The IgG titer was presented as binding antibody unit per mL (BAU/mL) according to the WHO international standard (NIBSC code 20/136), using Softmax Pro software (Version 5.4.1).

### Focus reduction neutralization test

Vero cells (#CCL-81, ATCC) were prepared in Dulbecco’s modified Eagle medium (DMEM; #11995065, Invitrogen) supplemented with 10% FBS and 1% Antibiotic-Antimycotic (#15240062, Gibco) and seeded at a density of 2.0 × 10^4^ cells/well in 96-well flat bottom plates (#167008, Thermo Scientific) and incubated overnight at 37°C with 5% CO2. Using DMEM containing 2% FBS, samples were serially diluted threefold, and SARS-CoV-2 stocks were diluted to 1.8 × 10^4^ PFU/mL. Serum was mixed with viruses (540 PFU/well) in U-bottom 96-well plates (#34196, SPL) and incubated at 37°C with 5% CO2 for 30 minutes. Vero cell plates prepared the day before were washed with serum-free DMEM, exposed to the virus-serum mixture (450 PFU/well), and incubated at 37°C with 5% CO2 for 5 hours. The cells were rinsed with PBS, fixed with 10% formalin (#HT501128-4L, Sigma-Aldrich), and permeabilized with ice-cold 100% methanol (#32213–1L, Sigma-Aldrich). The plates were washed with PBS and treated with a blocking buffer (PBS + 1% BSA (#A3803-100G, Sigma-Aldrich) + 0.5% goat serum (#Ab7481, Abcam) + 0.5% Tween-20 (#T9100-100, GenDEPOT)) for 30 minutes. Anti-SARS-CoV-2 nucleoprotein rabbit mAb (#40143-R001, Sino Biological) was diluted 3,000-fold in blocking buffer and added to the plates, followed by incubation for 1 hour at 37°C. The plates were washed with washing buffer (PBS + 0.5% Tween-20), and goat anti-rabbit IgG-HRP diluted 2000-fold in blocking buffer was added and incubated for another hour at 37°C. After rinsing the plates thoroughly, True Blue solution (#5510-0030, SeraCare) was added for staining. The plates were then washed with tap water before counting the foci using a cell imaging reader (Cytation 7, BioTek). Neutralizing antibody titers were determined using SoftMax Pro GxP software (Version 7.1.2).

### Antibody-dependent complement deposition

ADCD was performed as previously described (46). Red fluorescent NeutraAvidin-coated beads were coupled with biotinylated SARS-CoV-2 FS antigens. The antigen-coupled beads were incubated with diluted plasma samples (diluted 1:10 in PBS) at 37°C for 2 hours to form immune complexes and subsequently washed. RPMI 1640 medium (#11875119, Gibco) with 10% FBS (#16000044, Gibco) was used to reconstitute lyophilized guinea pig complement (#CL4051, Cedarlane). The reconstituted guinea pig complement (#CL4051, Cedarlane) was added to the washed immune complexes and incubated at 37°C for 50 minutes. Deposited C3 was detected using an anti-C3 FITC-conjugated goat IgG (#0855385, MP Biomedicals). After a 15-minute incubation for C3 immunostaining, the samples were fixed with 0.5% paraformaldehyde (#554655, BD) in PBS and measured to determine the GMFI of FITC C3 deposition via flow cytometry using iQue3 (Sartorius).

### Antibody-dependent cellular phagocytosis

ADCP was performed as previously described (47). Yellow-green fluorescent NeutraAvidin-coated beads were linked to biotinylated SARS-CoV-2 FS antigens. These antigen-bead combinations were exposed to serum diluted 1:200 in PBS at 37°C for 2 hours and then washed with PBS. Subsequently, the resulting immune complexes were mixed with 2.5 × 10^4^ THP-1 cells (#TIB-202, ATCC) per well in THP-1 medium (RPMI 1640 + 10% FBS + 1.14% Antibiotic-Antimycotic) and incubated overnight in a 37°C and 5% CO2 environment. The samples were fixed with 0.5% paraformaldehyde in PBS the following day and analyzed with iQue3. Phagocytic scores were calculated based on the percentage of THP-1 cells containing fluorescent beads multiplied by the number of beads phagocytosed by each cell (percentage of bead-containing cells × MFI bead-positive/1,000,000).

### Antibody-dependent neutrophil phagocytosis

ADNP was tested as described previously (48). Yellow-green fluorescent NeutraAvidin-coated beads were conjugated with biotinylated SARS-CoV-2 antigens. These antigen-bead complexes were then incubated with serum diluted 1:80 (or 1:40 for the Omicron BA.2 FS antigen) in PBS at 37°C for 2 hours, followed by washing with PBS. Subsequently, the immune complexes were combined with 5.0 ×10^4^ HL-60 cells (#CCL-240, ATCC) per well in HL-60 medium (RPMI 1640 + 11.4% FBS + 1.14% GlutaMax I (#35050–061, Gibco) + 1.14% Antibiotic-Antimycotic) and incubated for 1 hour in a 37°C and 5% CO2 incubator. HL-60 cells were previously differentiated in HL60 differentiating medium consisting of 0.91% DMSO (#D2650, Sigma-Aldrich) supplemented with 11.4% FBS and 1.14% GlutaMax I in RPMI 1640 6 days before sample analysis. After incubation, the neutrophils were stained with the CD11b antibody (#555388, BD BioSciences) diluted 1:1 in PBS. Following a 15-minute incubation, the cells were washed with PBS and fixed with 0.5% paraformaldehyde diluted in PBS. The percentage of antibody-mediated phagocytic activity was quantified using iQue3. Phagocytic scores were calculated as the percentage of neutrophils containing fluorescent beads multiplied by the number of beads phagocytosed by each neutrophil (percentage of bead-containing cells × MFI bead positive/1,000,000).

### Antibody-dependent natural killer cell activation

CD107a expression in NK-92 cells was measured as described previously (43, 49). Briefly, a 96-well ELISA plate (#439454, Thermo Scientific) was coated with 30 µg of SARS-CoV-2 FS antigens and incubated at 37°C for 2 hours. The coated antigens were blocked with 5% BSA in PBS and incubated overnight at 4°C. Serum samples, diluted 1:60 in PBS, were added to the wells and incubated for 2 hours at 37°C. The plates were then washed thoroughly with PBS. NK-92 cells (#PTA-8836, ATCC) were prepared by resuspending them in NK cell culturing media (#5150, StemCell) supplemented with 10% horse serum (#16050-122, StemCell), 0.02 µg/mL IL-2 IS (#130-097-744, Miltenyi Biotec), and 1% penicillin-streptomycin (#15140122, Invitrogen). For NK cell detection, anti-CD107a-PE-Cy5 antibody (#555802, BD BioSciences), brefeldin A (diluted to 5 mg/mL in DMSO, #B6542, Sigma), and GolgiStop (#554724, BD BioSciences) were added to the medium. NK-92 cells were seeded at a density of 5 × 10^4^ cells per well and incubated for 5 hours in a 37°C and 5% CO2 incubator. The cells were then transferred to a 96-well V-bottom plate, fixed, and permeabilized using a fixation/permeabilization kit (#554714, BD Biosciences). The fixed cells were analyzed using iQue3, with gates set on singlet and CD107a+ cells.

### Antibody and FcR profiling

Antigen-specific antibodies and FcR profiling were performed as previously described (48). Antibodies and FcRs specific to the four biotinylated SARS-CoV-2 antigens (FS, S1, S2, and RBD) were measured by attaching them to Luminex Magplex-Avidin microspheres. The microspheres were blocked and combined in assay buffer before seeding into 96-well U-bottom plates (#34196, SPL) at a density of 4,000 antigen-coupled Magplex microspheres per well. The serum dilution factors varied for each antibody isotype and FcR assay for optimization. Serum concentrations for each type were prepared in PBS as follows: for prototype SARS-CoV-2 antigens, IgG (1:640), IgG1 (1:320), IgG2 (1:160), IgG3 (1:320), IgG4 (1:320), IgM (1:80), IgA1 (1:160), FcγR2A (1:80), FcγR2B (1:80), FcγR3A (1:80), FcγR3B (1:160), and FcRn (1:80), and for Omicron BA.2 antigens, IgG (1:320), FcγR2A (1:80), and FcγR3B (1:80). The diluted serum was co-incubated with the beads for 2 hours on a plate shaker at 650 rpm. Thereafter, the plates were washed with 0.05% Tween in PBS. Isotype stains (Southern Biotech) were diluted in assay buffer at specific concentrations (IgG (#9040-09) = 1:200, IgG1 (#9054-09) = 1:1,000, IgG2 (#9070-09)  = 1:200, IgG3 (#9210-09) = 1:200, IgG4 (#9200-09) = 1:200, IgM (#9020-09) = 1:200, and IgA1 (#9130-09) = 1:200). FcR stains were prepared by tagging Streptavidin-PE (#PJ315S-1, Agilent) to FcRs obtained from Sino Biological (FcγR2A (#10374-H27H1-B), FcγR2B (#10259-H27H-B), FcγR3A (#10389-H27H1-B), FcγR3B (#11046-H27H-B), and FcRn (#CT071-H27H-B)) in assay buffer. The plates were then incubated at room temperature on a plate shaker at 650 rpm for an hour, washed with 0.05% Tween in PBS, and then read using iQue3. The results are presented as GMFI of phycoerythrin for each sample.

### Statistical analyses

All calculations and visualization were performed with SAS version 9.4, R version 4.3.3, and GraphPad Prism version 8.3.0. For statistical analyses, all values were log-transformed (log10) except for ADCP, ADNP and ADNKA. Statistical differences between the two groups in violin plots were calculated using a two-sided Mann–Whitney U test (significance levels: *P < 0.05, **P < 0.01, ***P < 0.001, and ****P < 0.0001). The solid lines in the violin plot indicate the 25th and 75th quartiles, and the dashed line indicates the median value.

To assess and compare the induced immune response across various dose groups and over different time points, a circular plot was constructed. Each value was normalized by subtracting the minimum value and then dividing them by the range, defined as the difference between the maximum and minimum values across all time points; $\frac{Value_{feature}-Min\;\left(All\;values_{feature}\right)}{Max\;\left(All\;values_{feature}\;\right)-Min\;\left(All\;values_{feature}\right)}$. sPLS-DA was conducted using the function “splsda” of R package “mixOmics” to select the features which are important to discriminate the group (50). We employed 10-fold cross-validation, and the procedure was repeated 10 times to ensure selecting the most effective combination of the components.

## SUPPLEMENTAL MATERIAL

Supplemental Figures msphere.00998-24-s0001.pdf Figures S1 to S5.

ASM does not own the copyrights to Supplemental Material that may be linked to, or accessed through, an article. The authors have granted ASM a non-exclusive, world-wide license to publish the Supplemental Material files. Please contact the corresponding author directly for reuse.
