# Supplementary material for: Dose-dependent serological profiling of AdCLD-CoV19-1 vaccine in adults
Source: mSphere. 2024 Dec 26;10(1):e00998-24. doi: 10.1128/msphere.00998-24 (PMC11774024; doi:10.1128/msphere.00998-24)
Supplement: Supplemental Figures — Figures S1 to S5. [file msphere.00998-24-s0001.pdf]

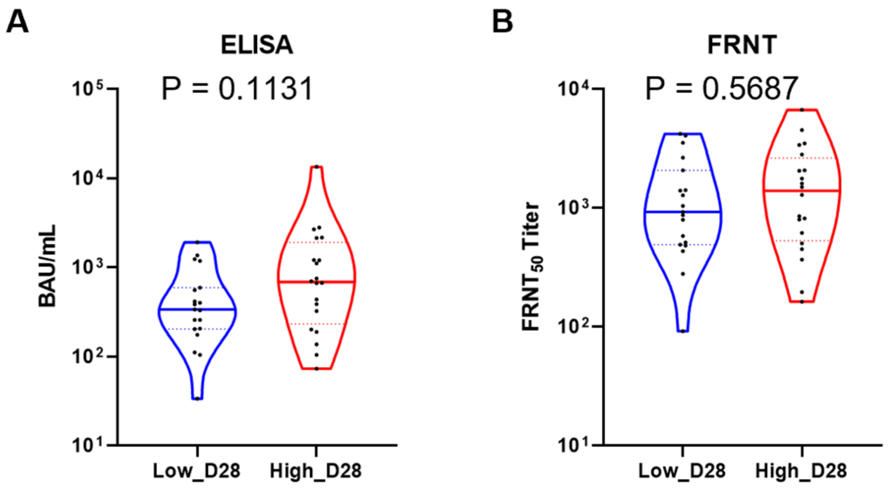

Figure S1. Anti-SARS-CoV-2 Spike IgG and neutralizing antibody titers of the two dose groups on day 28 post-vaccination.

Violin plots (representing medians, interquartile ranges [IQRs], minima, and maxima) showing the antibody levels in the low- and high-dose groups on day 28. Dots represent participants (n=20). (A) Anti-SARS-CoV-2 Spike IgG titers were measured as log10 binding antibody units/ml (BAU/ml) by ELISA. (B) Log10 titers of neutralizing antibodies against SARS-CoV-2 measured using the FRNT assay. Changes in antibody levels were compared using the Mann–Whitney U test. The p-values are shown in the figure.

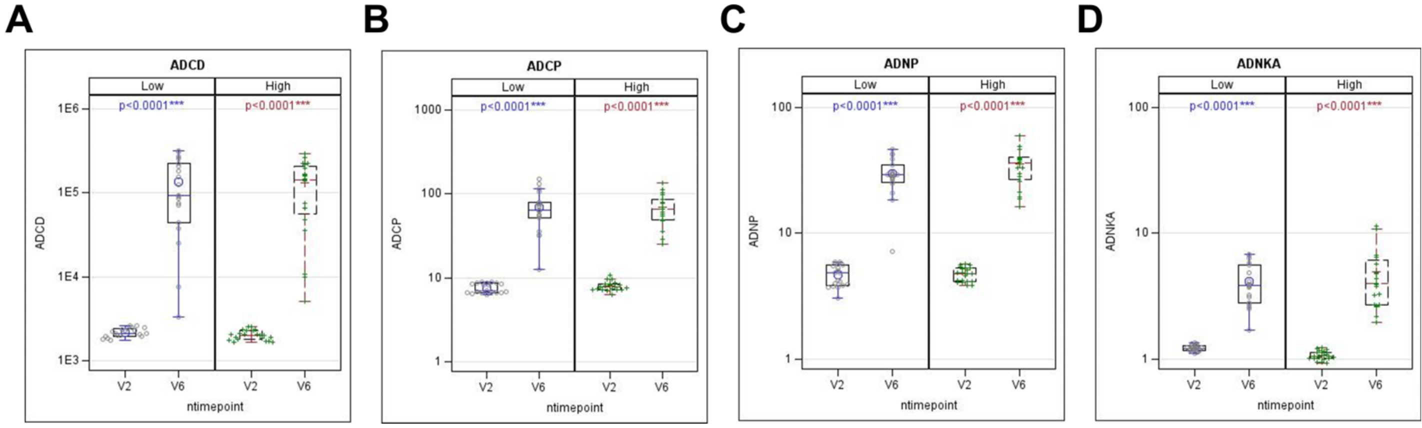

Figure S2. Effector functions across the two dose groups following vaccination.

Box plots (representing medians, means, interquartile ranges [IQRs], minima, and maxima) demonstrate the changes in the four FcR-mediated effector functions of the low- and high-dose groups on days 0 (V2) and 28 (V6). (A) Measurements are presented as GMFI (geometric mean fluorescence intensity) for C3 deposition, indicating antibody-dependent complement deposition (ADCD); (B) as phagocytosis scores for antibody-dependent cellular phagocytosis (ADCP) and (C) antibody-dependent neutrophil phagocytosis (ADNP); and (D) as the percentage of CD107a-positive cells, representing antibody-dependent NK-cell activation (ADNKA). Values on days 0 and 28 were compared using the Mann–Whitney U test. The p-values are shown in the figure.

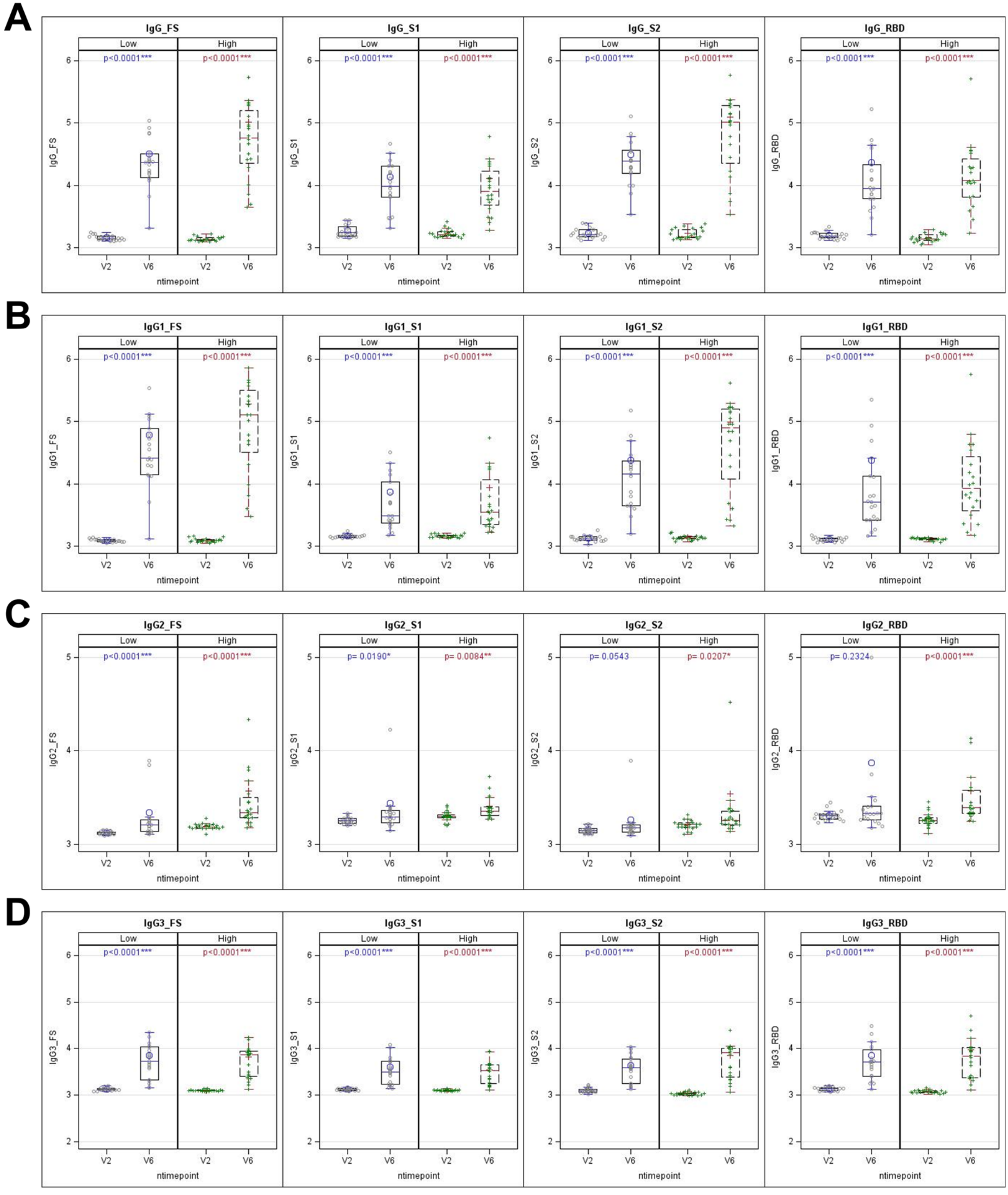
A
IgG_FS
IgG_S1
IgG_S2
IgG_RBD
B
IgG1_FS
IgG1_S1
IgG1_S2
IgG1_RBD
C
IgG2_FS
IgG2_S1
IgG2_S2
IgG2_RBD
D
IgG3_FS
IgG3_S1
IgG3_S2
IgG3_RBD
Low
High
p<0.0001***
p= 0.0190*
p= 0.0084**
p= 0.0543
p= 0.0207*
p= 0.2324
V2
V6
ntimepoint

E

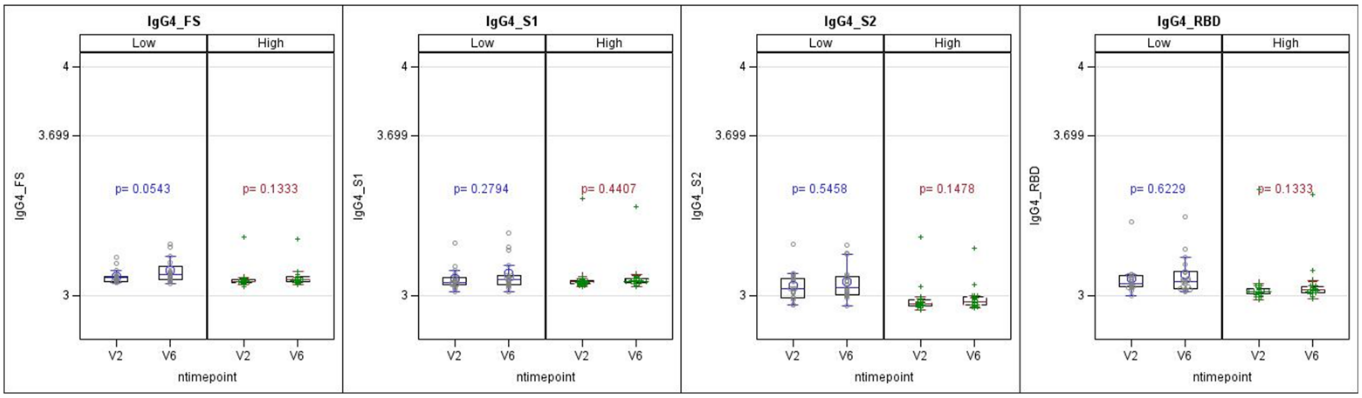

F

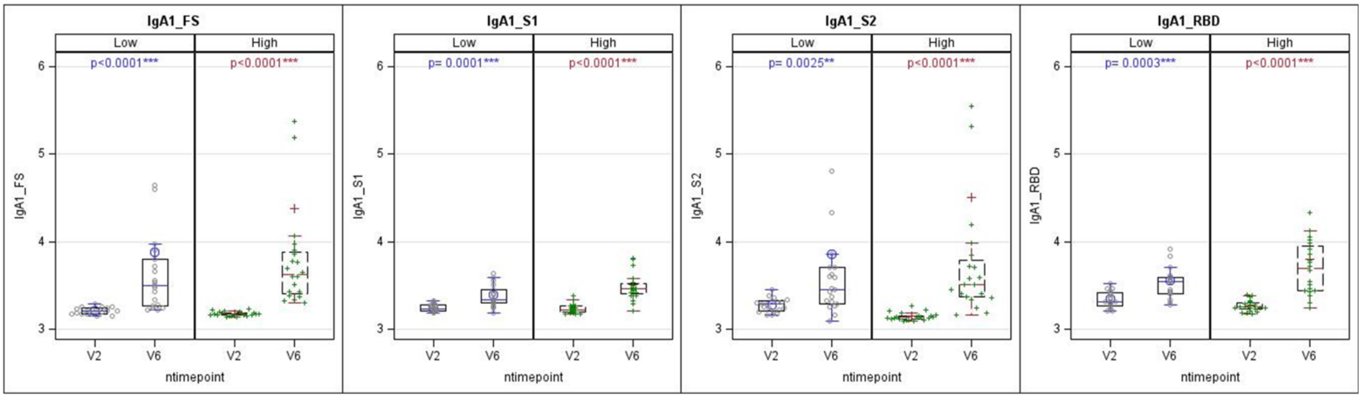

G

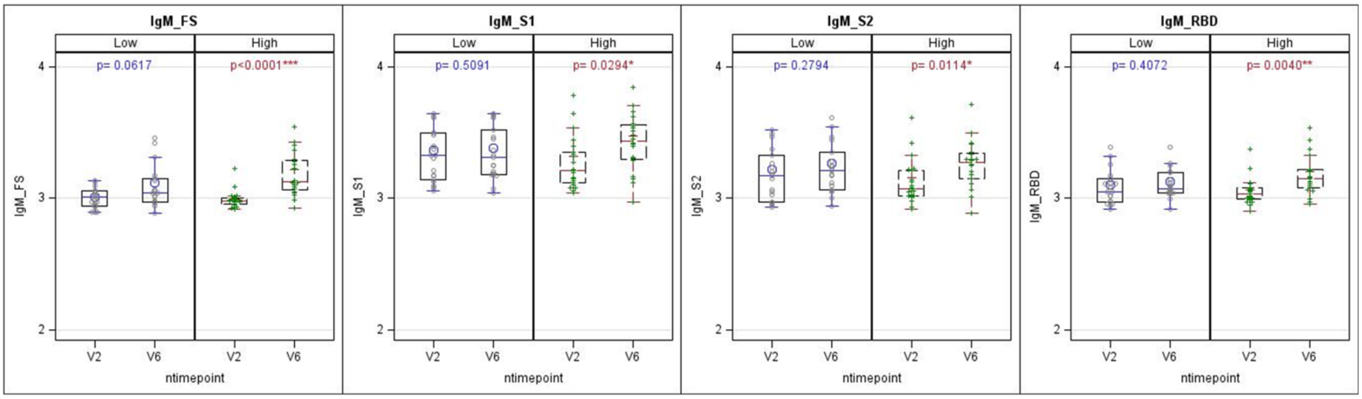

Figure S3. Antibody responses specific to prototype SARS-CoV-2 FS, S1, S2, and RBD across two dose groups following vaccination.

Box plots illustrating the antibody titers specific to prototype SARS-CoV-2 FS, S1, S2, and RBD in the low- and high-dose groups on days 0 (V2) and 28 (V6). Data are presented as log10 geometric mean fluorescence intensity (GMFI). The analyzed antibody isotypes and subclasses included (A) IgG, (B) IgG1, (C) IgG2, (D) IgG3, (E) IgG4, (F) IgA1, and (G) IgM. Changes in antibody levels were compared using the Mann–Whitney U test. The p-values are shown in the figure.

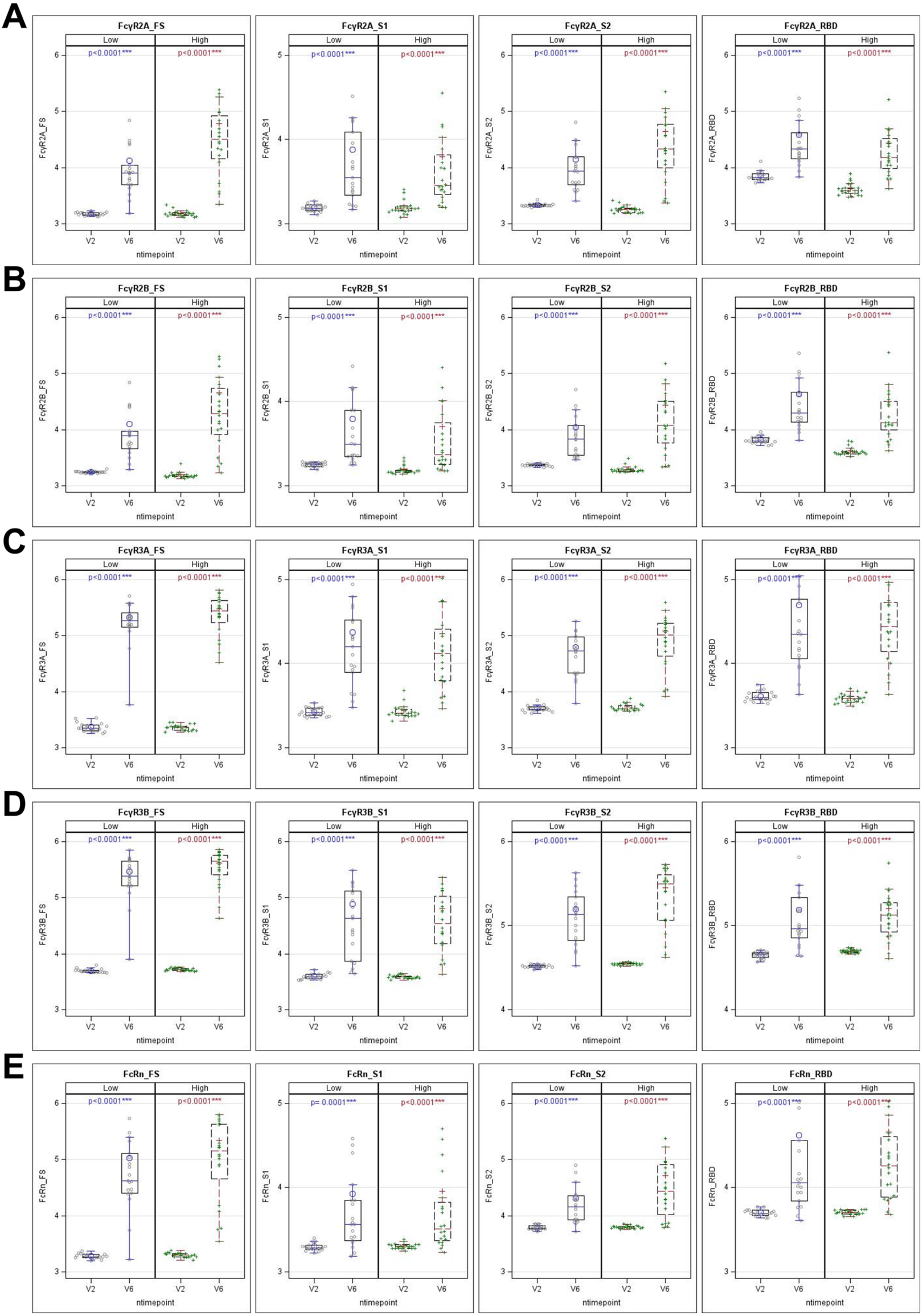
A
FcγR2A_FS
FcγR2A_S1
FcγR2A_S2
FcγR2A_RBD
B
FcγR2B_FS
FcγR2B_S1
FcγR2B_S2
FcγR2B_RBD
C
FcγR3A_FS
FcγR3A_S1
FcγR3A_S2
FcγR3A_RBD
D
FcγR3B_FS
FcγR3B_S1
FcγR3B_S2
FcγR3B_RBD
E
FcRn_FS
FcRn_S1
FcRn_S2
FcRn_RBD
Low
High
p<0.0001***
p= 0.0001***
V2
V6
ntimepoint

Figure S4. FcR binding responses specific to prototype SARS-CoV-2 FS, S1, S2, and RBD across the two dose groups following vaccination.
Box plots depicting the FcR binding responses specific to prototype SARS-CoV-2 FS, S1, S2, and RBD in the low- and high-dose groups on days 0 (V2) and 28 (V6). Data are presented as log10 geometric mean fluorescence intensity (GMFI). (A) FcγR2A, (B) FcγR2B, (C) FcγR3A, (D) FcγR3B, and (E) FcRn were analyzed. Changes in FcR levels were compared using the Mann–Whitney U test. The p-values are shown in the figure.

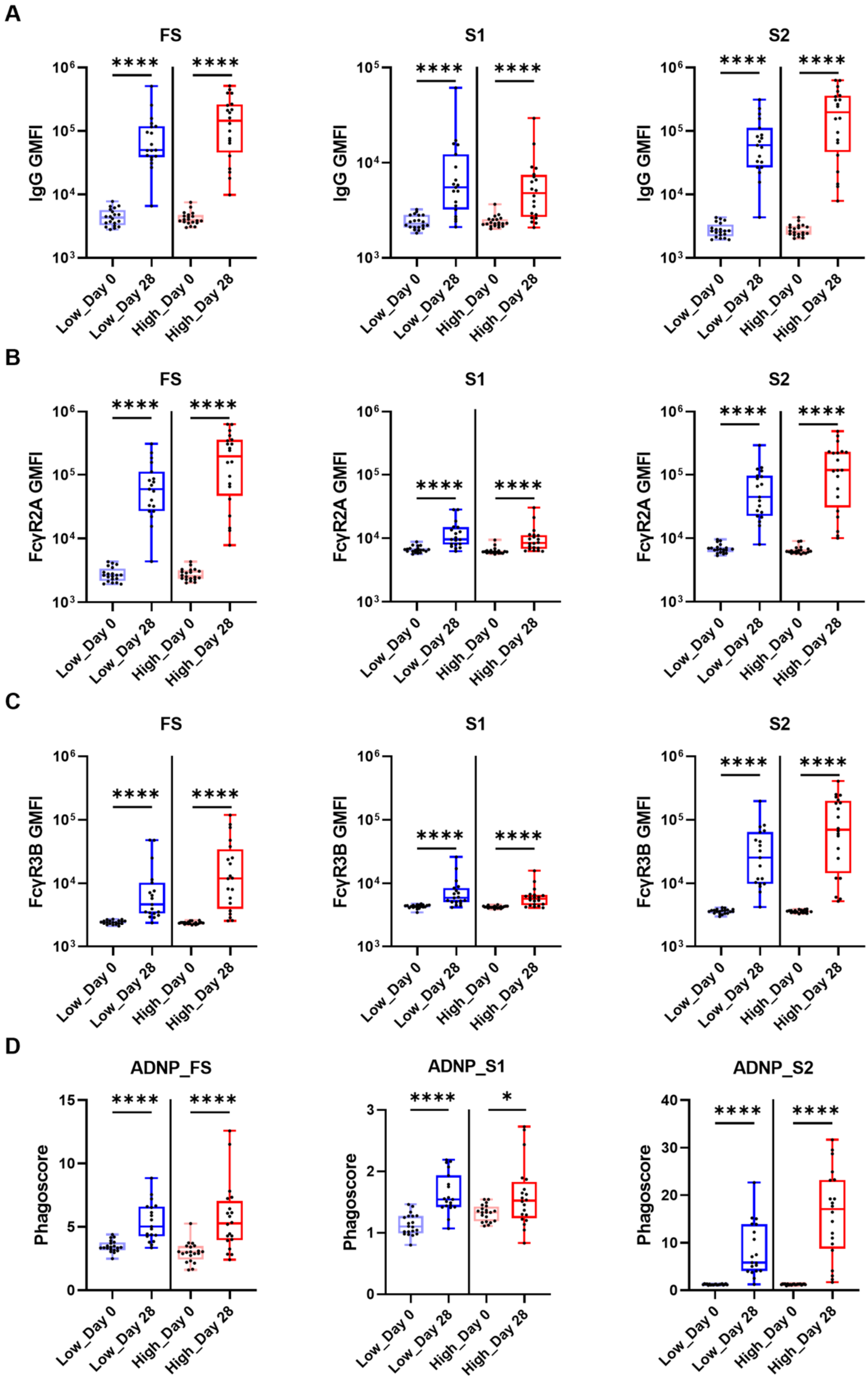

Figure S5. IgG, FcγR2A, FcγR3B, and neutrophil responses specific to Omicron BA.2 FS, S1, and S2 across the two dose groups following vaccination.
Box plots depicting (A) IgG titer, binding responses of (B) FcγR2A and (C) FcγR3B, and (D) ADNP to Omicron BA.2 FS, S1, and S2 in the low- and high-dose groups on days 0 and 28. Data for antibody and FcRs are presented as geometric mean fluorescence intensity (GMFI), and phagocytosis scores are shown for ADNP. Changes between pre- and post-vaccination were compared using the Mann–Whitney U test. The p-values are shown in the figure. *$P < 0.05$, ****$P < 0.0001$.
